# Psychiatry in the Middle East: the rebirth of lunatic asylums?

**DOI:** 10.1192/bji.2020.22

**Published:** 2021-02

**Authors:** Joelle M. Abi-Rached

**Affiliations:** MSc, MD, PhD, Postdoctoral Fellow, Society of Fellows in the Humanities, Columbia University and Invited Researcher, École Normale Supérieure, Paris, France. Email: joelle.abi-rached@ens.fr

**Keywords:** History of psychiatry, low- and middle-income countries, transcultural psychiatry, anthropology, economics

## Abstract

This article briefly assesses the historical trajectory of psychiatric institutions in the Middle East. It underlines a key observation: the persistence and expansion of psychiatric institutionalisation, specifically in the Arab world. In contrast to the deinstitutionalisation that eventually closed large psychiatric hospitals in the 1960s and 1970s, notably in Europe and North America, psychiatric hospitals have continued to grow in size in the Arab world. This absence of deinstitutionalisation marks a major departure from how psychiatry developed in the West, which is worth reflecting on if we are to understand the current crumbling infrastructure of in-patient psychiatric facilities in the Arab region.

On 17 February 2019 a health worker at Al-Fanar, a psychiatric hospital in south Lebanon, reported to the media the indecent and inhumane conditions in which patients had been living; they lacked heating, food, medicine and basic hygiene.^1^ Her description could well have been made in the 19th century, when visitors to ‘Oriental lands’ often decried the barbaric treatment of the insane.^2^ The exposé caused nationwide outrage, prompting the closure of the hospital. At the time of writing, the case is still in court.

## A crumbling infrastructure

When I visited Al-Fanar in 2012, there was no visible proof of filth or deliberate neglect although the hospital facilities looked rudimentary and the dormitories were crowded and bleak. In 2017, I witnessed likewise the degrading state of the main psychiatric hospital at Blida, outside Algiers, where Frantz Fanon, the influential Martinique psychiatrist, taught and worked in the mid-1950s.^3^ Once the pride of the colonial Algerian School of Psychiatry, the Hôpital Psychiatrique de Blida-Joinville is today underfunded and neglected; its wards look like prison cells, where patients behind bars beg for money and cigarettes while a dusty portrait of Fanon greets the visitors.

While mental illness continues to be stigmatised in the Middle East,^4^ what is more flagrant is the crumbling in-patient psychiatric care that is increasingly visible across the Arab region, partly due to neglect and partly to war. A 2012 report on human rights and mental health in Morocco deplores the lack of adequate care, derelict buildings, scarce equipment, shortage of staff, outdated laws, overcrowded facilities and serious human rights violations.^5^ The ongoing war in Syria has exacerbated the precarious state of the psychiatric infrastructure. Patients have been killed and various psychiatric hospitals, such as the Ibn Sina psychiatric hospital (465 beds) near Damascus, have been severely damaged by shelling.^6^ In the Iraqi context, one can tell a similar story of neglect, lack of psychiatric personnel, overcrowded facilities and decaying infrastructure, all of which are in great part the consequence of now two decades of conflict and war, displacement and terror.^7^

In retrospect, the downfall of Al-Fanar reflects a larger story of state failure, ossified institutions, collapsed infrastructure, aborted modernisation projects and lack of visionary alternatives across most of the Middle East and North Africa (MENA) region.

## The rise and fall of lunatic asylums

The long history of mental healthcare in the region has seen repeated cycles of good care that eventually break down. In the 10th century, the insane were treated in a general hospital called *bimaristan* (Persian for ‘house of the sick’), where their care consisted in rest, diet, baths, purgatives and music therapy. This complex institution, however, declined during the Ottoman Empire and was eventually supplanted by the European lunatic asylum.

In 19th-century Constantinople or Cairo, foreign doctors were called upon to revamp the main public asylums. The Italian alienist Luigi Mongeri was named superintendent of the Süleymaniye lunatic asylum in 1856, and the British physician Frank Sandwith began to reform Cairo's ‘Abbasiyya asylum in 1884 as Egypt passed from khedival to British rule. In the Near East, the Lebanon Hospital for the Insane (‘Aṣfūriyyeh), which was established in 1896 in Mount Lebanon, was the only voluntary mental hospital when it opened in 1900. Unlike Al-Fanar hospital, however, ‘Aṣfūriyyeh's closure in 1982 was mainly due to the long and costly Lebanese civil war.^8^

In Europe and North America, psychiatric hospitals kept growing in size and the cost of their maintenance kept soaring until the mid-20th century, when a confluence of factors – the discovery of powerful anti-psychotics, a general discontent with these institutions as places of confinement and a strategy to move to community healthcare centres in an attempt to reduce state expenditure – brought their demise in a process that came to be known as deinstitutionalisation.^9^

## The resilience of institutionalisation

As large psychiatric hospitals in the West started to close down in the 1960s, in-patient care shifted to psychiatric units within general hospitals and community-based residential care facilities. In stark contrast, mental hospitals continued to grow in size in the Middle East. Today, Egypt, Lebanon, Algeria and Iraq host some of the largest psychiatric hospitals in the world; Egypt's ‘Abbasiyya, which in 1889 had 300 beds, has today around 1500 beds, Al-Rashad Mental Hospital (al-Shamma'iyya), which was founded in the early 1950s in Baghdad with originally 400 beds, now has more than 1300 beds, Lebanon's Hôpital Psychiatrique de la Croix (Dayr al-Salib), which started in 1919 as a modest asylum for elderly priests, has more than 1100 beds, and Algeria's Blida-Joinville psychiatric hospital, which opened in 1938 with 1200 beds, has today an impressive capacity of 2200 beds.

High-income countries (globally) tend to have many more psychiatric beds (over 50 per 100 000 population) than low- and lower-middle-income countries (fewer than 7 per 100 000). The majority of these beds, however, tend to be in psychiatric units within general hospitals and/or in community-based residential facilities rather than in mental hospitals.^10^ According to the World Health Organization (WHO), this configuration of mental health in-patient care is one indication of successful deinstitutionalisation.^11^

**Fig. 1:**
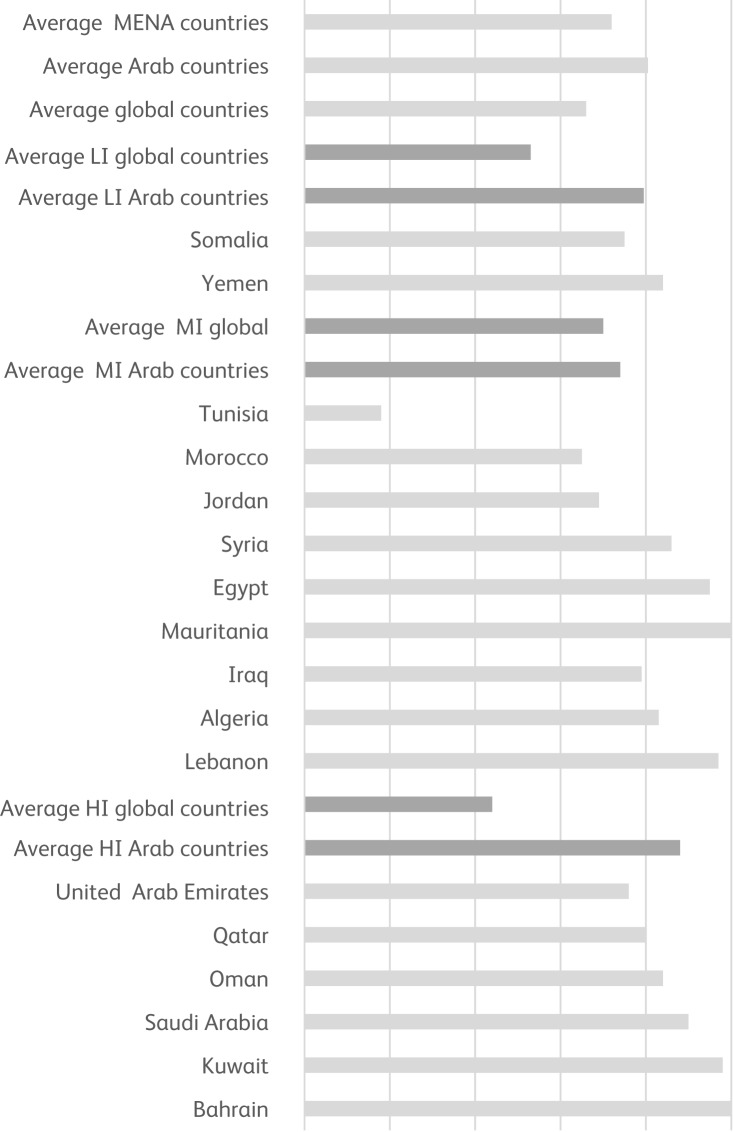
Proportion of psychiatric beds in mental hospitals in the MENA region and globally, based on data from the WHO's Mental Health Atlases for 2011^12^ and 2017.^10^ The data for Libya and Sudan are not reliable and are therefore not included. HI, high-income; MI, middle-income; LI, low-income; MENA, Middle East and North Africa.

We see the opposite development in the Middle East (Fig. 1). Although the average for the MENA region in terms of institutionalisation pattern (beds in mental hospitals as a proportion of all psychiatric in-patient beds) is not dramatically worse (72%) than the global average (66%), the difference is more substantial for Arab countries (which have 81% of beds in mental hospitals, on average). A comparison of Arab countries with their global income comparators shows a consistent pattern, including both high-income countries (88% *v*. 44%) and low-income countries (80% *v*. 53%) in the region. Interestingly, the middle-income group average (countries such as Iraq, Syria, Egypt and Lebanon) is similar to its global middle-income comparator (74 and 70% respectively).

The relatively good average performance of the middle-income group hides, however, great disparities. Some countries, such as Tunisia, are successfully implementing WHO-recommended policies. But Tunisia is the exception in the Arab world, in that it has markedly downsized its single mental hospital and created new psychiatric units in general hospitals, although it still lacks adequate mental health resources as well as community mental health services.^13^ Other countries have fallen behind. For example, Egypt has a far higher proportion of psychiatric beds in mental hospitals than in general hospitals. Some countries in the middle-income group (such as Iraq in 2004 or Jordan in 2011) launched national mental health policies and produced plans to build new community-based psychiatric facilities, based on the WHO Mental Health Gap Action Programme.^14^ Unfortunately, in Iraq, perhaps because of continuous political instability and civil strife, efforts to improve services and care seem to have ceased.^15^ In Jordan, mental health resources remain scarce and centralised, despite important gains.^16^

In general, the non-Arab MENA countries, namely Israel (which has the highest number of psychiatric beds per 100 000 population in the region), Turkey and Iran, fare much better than their Arab counterparts. In these three countries, the mental health infrastructure is comparable to that in Europe or the USA, which have 35 and 37% of psychiatric beds in general hospital units and/or community-based facilities respectively. It is the performance of these three countries that pushes the MENA average down to 72% overall.

What can explain the lack of deinstitutionalisation in that part of the world? The WHO often highlights the lack of both community healthcare alternatives and comprehensive mental health strategies. But political myopia and instability are probably what makes deinstitutionalisation difficult to implement. There are also other considerations, such as increasing urbanisation alongside a shrinking household size (making it cheaper to maintain mentally ill relatives in such institutions, which are generally public and therefore free of charge). Finally, one factor that is never mentioned, perhaps because it is too polemical, and yet is perhaps as significant as the previous ones, concerns the lack of debate about the hegemony of psychiatrists and the exercise of psychiatric power, particularly during the crucial period of the 1960s–1970s, but even today; this situation contrasts with many Western countries, which saw the emergence of vocal criticisms of contemporary psychiatric practices, particularly the use of ‘asylums’ to detain people with mental illnesses.

## The need for self-reflective critiques

In Europe and North America, asylum exposés in the 1950s and 1960s had led to furious self-reflective critiques of contemporary psychiatric practices. Many prominent, if controversial, psychiatrists, such as R.D. Laing, Thomas Szasz and Franco Basaglia, provocatively questioned the underlying assumptions and rationales of psychiatry. While these psychiatrists, as well as the anti-psychiatry movement more broadly speaking, had their detractors and critics (their arguments and attitudes have, for some, become passé), the outcome was a profound rethinking of psychiatric practice and reconceptualisation of mental health.^17^

In the Arab world, in contrast, such exposés have tended to fall on deaf ears. The Arab region has had several prominent psychiatrists since the death of Fanon (who, interestingly, embraced rather than rejected psychiatry while using it to denounce colonialism as dehumanising, traumatic and alienating). But these psychiatrists tended to be either great reformers and administrators (bringing more humanity and openness to an otherwise stigmatised institution) or informers, creatively drawing on their practice to understand the roots of war-related trauma as well as of religious extremism. What is missing, however, is a candid critique of psychiatry itself, of its problematic proximity to power structures and of its reductionist and essentialising discourse, the way this critique was articulated in the West in the 1960s and 1970s. The reasons are complex, but they need to be addressed if we are to grasp the resilience of institutionalisation in that part of the world.

To go back to the title of this article, are we witnessing in the Middle East the rebirth of ‘lunatic asylums’ – these large mental institutions that marked the long 19th century up until the mid-20th century? The answer in short is ‘yes’ for the Arab world, where mental hospitals continued to grow in size despite their decline in Europe and North America. Iran, Turkey and Israel are the big regional exceptions, along with Tunisia. Although some mental hospitals in the Arab world have had to close (because of lack of funds, mismanagement or war), the vast majority of them continue to operate under rudimentary conditions, bringing to a full circle the image of the mental hospital as a dark place of incarceration and confinement rather than cure and hope.

## Supplementary material

For supplementary material accompanying this paper visit https://doi.org/10.1192/bji.2020.22. click here to view supplementary material
